# A Comprehensive Review and Insights into the New Entity of Differentiated High-Grade Thyroid Carcinoma

**DOI:** 10.3390/curroncol31060252

**Published:** 2024-06-09

**Authors:** Agnes Stephanie Harahap, Regina Stefani Roren, Shofiyya Imtiyaz

**Affiliations:** 1 Department of Anatomical Pathology, Universitas Indonesia, Dr. Cipto Mangunkusumo Hospital, Jakarta 10430, Indonesia; reginastefaniroren@gmail.com; 2 Faculty of Medicine, Universitas Indonesia, Jakarta 10430, Indonesia; shofiyyaimtiyaz@gmail.com

**Keywords:** high-grade, poorly differentiated, thyroid carcinoma, well-differentiated

## Abstract

Differentiated high-grade thyroid carcinoma (DHGTC) is a new subset within the spectrum of thyroid malignancies. This review aims to provide a comprehensive overview of DHGTC, focusing on its historical perspective, diagnosis, clinical characteristics, molecular profiles, management, and prognosis. DHGTC demonstrates an intermediate prognosis that falls between well-differentiated thyroid carcinoma and anaplastic thyroid carcinoma. Previously unenumerated, this entity is now recognized for its significant impact. Patients with DHGTC often present at an older age with advanced disease and exhibit aggressive clinical behavior. Molecularly, DHGTC shares similarities with other thyroid malignancies, harboring driver mutations such as *BRAF*V600E and *RAS*, along with additional late mutations. The unique behavior and histologic features of DHGTC underscore the necessity of precise classification for prognostication and treatment selection. This highlights the critical importance of accurate diagnosis and recognition by pathologists to enrich future research on this entity further.

## 1. Introduction

The recently updated World Health Organization (WHO) 2022 classification of Endocrine and Neuroendocrine Tumors added a new entity to the malignant thyroid neoplasm’s main group of classifications, the high-grade follicular-cell-derived non-anaplastic thyroid carcinoma [1]. According to the most recent recommendation, the additional differentiated high-grade thyroid carcinoma (DHGTC) stands along with poorly differentiated thyroid carcinoma (PDTC) in the group with intermediate prognosis, separated from the well-differentiated and undifferentiated type of thyroid neoplasms [1,2,3]. With this new guideline, pathologists were expected to comply and interpret these new criteria into practice, to understand the underlying pathology better, and the diagnostic, prognostic, and clinical significance of thyroid malignancies.

**Figure 1:**
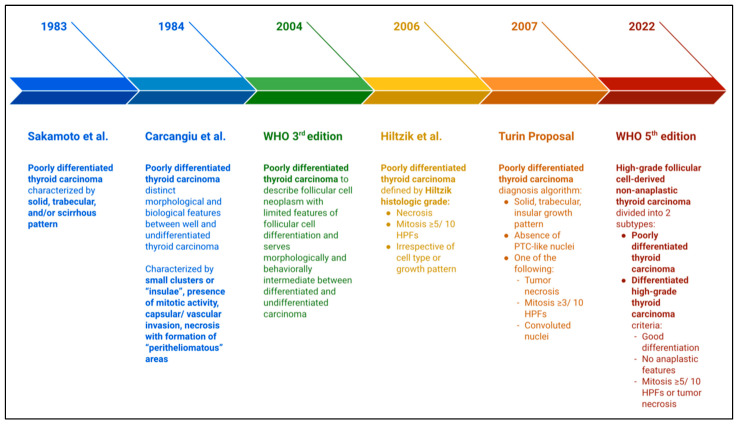
Evolution of high-grade follicular-cell-derived non-anaplastic thyroid carcinoma diagnosis and classification [1,4,5,6,7,8].

Forty years before the release of this classification, the concept of another type of thyroid carcinoma that stands in between the well-differentiated and undifferentiated carcinomas had been proposed, discussed, and modified into classifications that can be accepted among physicians and pathologists worldwide (Figure 1) [4]. Started in 1983 and 1984, Sakamoto et al. [5] and Carcangiu et al. [6], suggested a group of thyroid carcinomas classified by their growth pattern (solid, trabecular, or “scirrhous”) and necrosis/mitotic activity as a poorly differentiated carcinoma, respectively [5,6]. In 2004, the WHO made the term PDTC official as a new entity of thyroid neoplasm, that is otherwise still controversial and inconvenient to use [7]. Therefore, the Turin proposal was made in 2006 involving Japanese, American, and European pathologists to reduce bias and confusion among pathologists, which then produced the renowned algorithm of PDTCs diagnostic criteria. The diagnosis was made by considering the growth pattern, nuclear features, mitotic activity, and necrosis. The criteria for PDTC from Turin consensus were as follows: first, solid, trabecular, or insular growth pattern; second, absence of papillary thyroid carcinoma (PTC)-like nuclei; and third, featured at least one of the following: convoluted nuclei, mitotic index ≥3/10 HPFs, and/or tumor necrosis. This proposal was then released in 2007 [4].

Also in 2006, in New York City, USA, Hiltzik et al. [8] from Memorial Sloan-Kettering Cancer Center, conducted a study of PDTC from the cytomorphological perspective, accounting for the tumor necrosis and mitosis number of more than equal to 5 per 10 high-power fields (HPF), which is believed to be a more homogenous criteria than the previous suggestions. Hiltzik’s histological grade (HHG) defined PDTC irrespective of growth pattern and cell type, including PTC-like nuclei. In this study, it was proven that with these criteria, the PDTC group showed an intermediate prognosis of 60% of the 5-year overall survival (OS) compared to the well-differentiated (98% of the 10-year OS) and undifferentiated/anaplastic (0% of the 5-year OS) [8]. Moreover, both criteria, PDTC by HHG and PDTC by growth pattern, were compared and presented a significant difference in OS and disease progression, where the first group was more homogenous and had a worse prognosis. This concludes the previous hypotheses that histologic grade had a significant role as a prognostic value in thyroid carcinoma, specifically PDTC [9,10].

A follow-up study by Gnemmi et al. [9] from France was published in 2014, analyzing the implementation of both methods to date. The issue was to help decide which criteria were better used for diagnosing PDTC without over- or under-diagnosing thyroid neoplasm. From this study, both criteria fit to predict intermediate prognosis for thyroid carcinomas of follicular origin, except convoluted nuclei in Turin’s proposal which was proven to have a weak prognostic value [9]. However, they believe that the overlapping criteria should be further re-classified for a more uniform implementation among physicians [8]. On the note, Turin’s criteria are acceptable for diagnosing PDTC, including the architectural de-differentiation, but a different classification should be made to cover the HHG’s high-grade features of similar prognosis by including the well-differentiated feature, such as PTC-like nuclei [11].

Further study by Wong et al. [12] suggests a similar result, that PTC with high-grade features should not be set aside, as they possess distinct characteristics in pathologic, molecular, and clinical features. They appear to be more aggressive than PDTC by WHO criteria, in terms of capsular and vascular invasion, metastasis, and later survival rate [12,13]. Recent studies have indicated that minor components of high-grade features, such as necrosis and poor differentiation, may significantly impact disease prognosis [7,14,15,16]. Redefining the criteria is important to detect such disease progressiveness and prevent underdiagnosing patients with thyroid neoplasms as a well-differentiated one. In 2022, the dilemma came to light by the distinction of DHGTC as a new entity of thyroid neoplasm [1]. In this study, we aim to discuss further and review the newly added subtype and how it may differ from one another.

## 2. The New Classification of Malignant Thyroid Carcinoma

**Figure 2:**
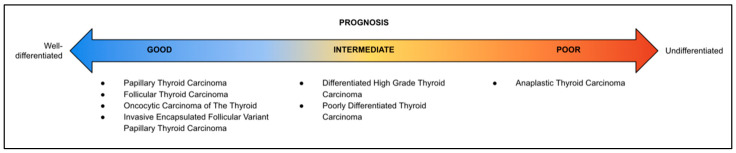
Classification of malignant thyroid follicular-cell-derived neoplasms related to differentiation and prognosis.

In the latest update of the 5th edition of WHO 2022 Classification of Endocrine and Neuroendocrine Tumors, there were several changes, including the re-classification of several tumor subtypes. Thyroid follicular-cell-derived neoplasms were divided into benign lesions, low-risk neoplasms, and malignant thyroid neoplasms. Malignant thyroid neoplasms are further divided into six types of tumors, which are follicular thyroid carcinoma (FTC), papillary thyroid carcinoma (PTC), invasive encapsulated follicular variant papillary thyroid carcinoma (iefvPTC), oncocytic carcinoma of the thyroid (OCA), high-grade follicular-cell-derived non-anaplastic thyroid carcinoma, and anaplastic thyroid carcinoma (ATC). These tumor types were classified morphologically by their biologic behavior, molecular alterations, and prognosis (Figure 2) [1,2,3,13]. Differentiated high-grade thyroid carcinoma was previously PTC, FTC, and OCA with high-grade features [7]. This newly added subtype differs from PDTC in terms of its predominantly well-differentiated follicular-derived carcinoma, nuclear features, and growth pattern while sharing a similar intermediate prognosis [2,3].

A meta-analysis by Poma et al. [17] states that DHGTC is more prevalent in FTC (with a similar proportion of minimally invasive and widely invasive types) and in some aggressive subtypes of PTC, including solid/trabecular, tall cell, and hobnail. Classically, these PTC subtypes exhibit a higher incidence of adverse clinical features such as extrathyroidal extension, lymph node involvement, distant metastases, and an increased risk of recurrence. Although found in a small proportion of tumors, accounting for less than 11% of cases, these aggressive clinical behaviors are notable even when they manifest in a limited proportion of the neoplastic tissue [18]. High-grade features may further escalate the aggressiveness of these tumors. The Ki67 labeling index may hold value in objectively identifying high-grade behavior among histological groups not fitting the DHGTC criteria [19]. It should also be noted that the classic, diffuse sclerosing, and follicular subtypes of PTC exhibit a lower likelihood of reclassification as DHGTC [17].

## 3. Diagnosis

Differentiated high-grade thyroid carcinoma is defined as an invasive tumor of PTCs/FTCs/OCAs with ≥5 mitoses per 2 mm^2^ and/or tumor necrosis with no anaplastic features. Whereas PDTC is defined by the presence of a solid/trabecular/insular growth pattern, in the absence of conventional nuclear features (such as PTC-like nuclei), with at least one high-grade feature (convoluted nuclei, ≥3 mitoses/2 mm^2^, and/or tumor necrosis) [2,3,20]. The different criteria represent distinct mutagenic changes, pathologic features, and clinical behavior in both PDTC and DHGTC (Table 1), highlighting the high-grade features (necrosis and mitosis) and inclusion of PTC-like nuclei in DHGTC diagnosis [3,8,9]. Although in some cases tumors might have mixed features of differentiated and poorly differentiated components, all findings should be recorded [20].

### 3.1. Macroscopic Features

**Figure 3:**
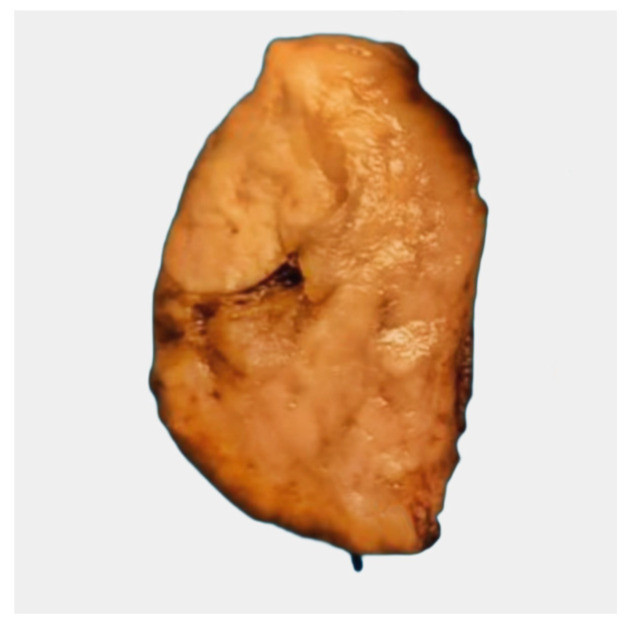
The gross examination of the tumor revealed a large solid mass with a white-brown color.

Macroscopically, PDTC, and DHGTC can form complete or partial encapsulation to gross infiltration and extrathyroidal extension. Macroscopically, the tumors appear as a large solitary mass, with almost even laterality between the left and right lobes. The average size ranges from 4.05 to 6.5 cm [9,16,21,22,23,24]. The cut surface often revealed a firm, white-tan texture, with necrosis serving as an important diagnostic indicator (Figure 3) [1].

### 3.2. Microscopic Features

Since the introduction of the new criteria, only a limited number of studies have been published that present data on DHGTC cases and their clinical significance, as detailed in Table 2.

#### 3.2.1. Necrosis

**Figure 4:**
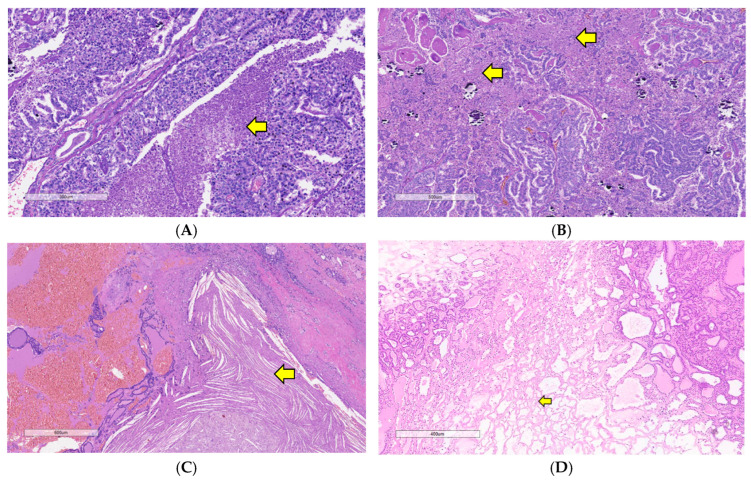
(**A**) Comedo-necrosis characterized by necrosis and nuclear debris (yellow arrow); (**B**) Extensive necrosis in high-grade differentiated carcinoma arising from papillary thyroid carcinoma (yellow arrows); (**C**) Post-fine needle aspiration biopsy showed an area of necrosis with hemorrhages and cholesterol cleft (yellow arrow). This finding shall not be mistaken for a tumor necrosis; (**D**) The necrosis should not be mistaken for infarct necrosis as shown by the arrow (hematoxylin and eosin).

High-grade features marked by tumor necrosis and/or high mitotic count have been a strong negative prognostic factor in thyroid neoplasms, especially tumor necrosis [8,9,25,26]. Types of necrosis that are included as a high-grade feature are also called “fresh”, “spontaneous”, “comedo”, or “true” tumor necrosis (Figure 4A,B) [8,25,26]. Necrosis appears as a product of mutagenic changes as the disease progresses (early and late event changes) as a sign of aggressive tumor activity [8]. The characteristics of tumor necrosis include distinct follicular cell differentiation, signs of degenerating cytoplasm, destructive fragmentation of nuclear debris, and irregular distribution of chromatin, accompanied by ghost cells [2,8,26,27]. However, it should not be confused with another type of necrosis such as infarct-necrosis which is influenced by fine needle aspiration procedure, which can be detected from the presence of hemorrhage, fibrosis, granulation tissue, calcification, and even an identifiable needle tract (Figure 4C,D) [26,27,28]. At first, in the MSKCC study of high-grade features in PDTC, tumor necrosis was divided based on the percentage area of necrosis, focal (≤5%) and extensive (>5%). However, it is proven insignificant and not mandatory in diagnosing DHGTC, because even a small area of necrosis has been correlated with a worse prognosis [8,24]. Recent studies only categorized necrosis as present or absent, which means any area of necrosis is accountable for making a diagnosis of PDTC and DHGTC [16,29,30].

#### 3.2.2. Mitosis

**Figure 5:**
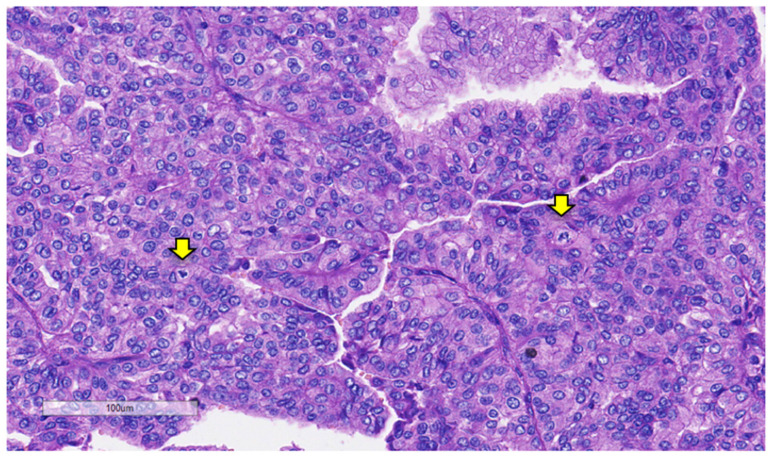
Mitotic figures are marked by arrows (hematoxylin and eosin).

Although the significance of the mitotic count in thyroid neoplasm was previously unclear, the mitotic count has been mentioned long before the diagnostic criteria were made. It was previously classified as an “atypical” feature of thyroid neoplasm [29,31]. Since the majority of capsulated and differentiated carcinoma are indolent in nature and harbor very low mitotic activity, the increasing mitotic counts have gathered interest. Studies showed a significant role of the increasing mitotic counts to the disease progression and prognosis. It is later included in the criteria to classify thyroid neoplasm [16,29,31]. Turin’s proposal defined mitosis in PDTC by ≥3/10 HPFs, whereas MSKCC criteria defined mitosis in PDTC by ≥5/10 HPFs, which the latter then adopted for DHGTC diagnosis criteria [1,2,3,4,8]. However, a study by Thomson et al. [16] suggests that necrosis is superior to mitotic count in the study cases, and so mitotic count of 4/mm^2^ or 5/mm^2^ did not show a significant difference [16]. Mitotic counts were scored in a more focused method, starting from the area with the highest mitotic activity, which is also called the “hotspots” [24,26]. The new WHO recommendation also suggests a change in the counting method; a standard 2 mm^2^ counting area is preferred rather than HPFs (2.4 mm^2^). This method is considered a more objective and applicable resource for both microscopic and digital images (Figure 5) [2,25]. Uniformity in the criteria for mitosis count cutoffs in the classification of PDTC and DGHTC is warranted. Further study is necessary to eliminate confusion and establish standardized thresholds.

#### 3.2.3. Growth Pattern

**Figure 6:**
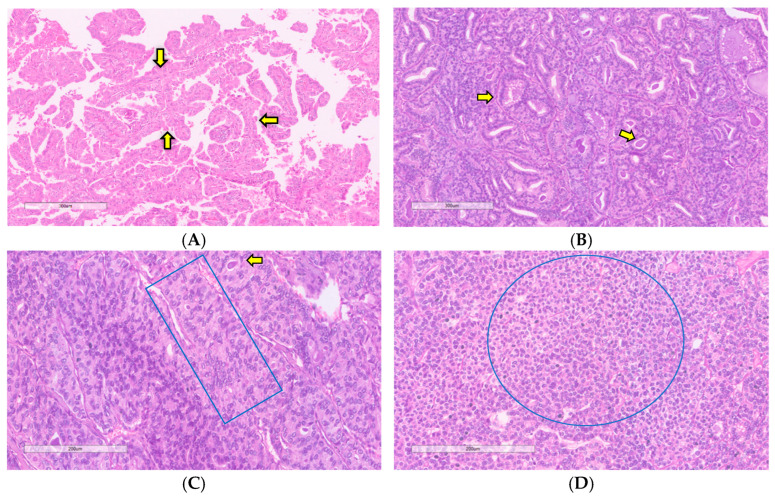
Growth patterns in thyroid neoplasm. (**A**) Papillary (arrow); (**B**) Follicular (arrows); (**C**) Trabecular (rectangle) and microfollicular (arrow); (**D**) Solid (circle) (hematoxylin and eosin).

Growth pattern is one of the differentiating categories between PDTC and DHGTC. Since 1984, Sakamoto et al. [5] have been suggesting a growth pattern as a marker of poor differentiation that defines the more aggressive behavior of PDTC. Along the way, Volante et al. [32] proved the extent of solid/trabecular/insular growth patterns does not significantly affect disease progressiveness. Therefore, the Turin algorithm 2007 and current WHO recommendation suggest a diagnosis of PDTC only based on the presence of solid, trabecular, or insular growth patterns, irrespective of the areas involved [1,4,33]. Solid patterns are defined by areas lacking follicular or papillary growth patterns, without nests or islands of cells [3,8,32,34]. Trabecular patterns appeared as elongated cords or ribbons of tumor cells, sometimes resembling a fence-like structure. Insular and solid patterns appear as islands or nests of tumor cells, surrounded by a thin layer of fibrovascular stroma, small vessels, and clefts (Figure 6) [6,16,32,35,36]. However, in both PDTC and DHGTC, patterns of follicular and papillary carcinoma were still commonly found, as a marker of good differentiation, in a clinically more aggressive thyroid carcinoma. Therefore, the diagnosis of DHGTC made it relevant to represent those groups irrespective of growth pattern.

#### 3.2.4. Nuclear Features

Nuclear features in PDTC and DHGTC might have several differences in characteristics. The presence of convoluted nuclei in PDTC hold diagnostic significance, whereas PTC-like nuclei are included in the DHGTC subtype [1,2,3,4]. In PDTC criteria, both the common nuclear features of papillary carcinoma and convoluted nuclei are included. Convoluted nuclei appear slightly smaller and darker than the typical nuclei of papillary carcinoma, with irregular nuclear contours and membrane convolutions [4]. However, the molecular background and prognostic significance become questionable in some studies, as they show conflicting results. A study by Gnemmi et al. [9] shows convoluted nuclei are a weak prognostic factor of PDTC. Another study by Asioli et al. [34] also shows a positive prognostic effect from the convoluted nuclei feature [9,34]. Uniform diagnosis of convoluted nuclei might also become a challenge among pathologists, resulting in insufficient data and needing further adjustment to utilize more standardized criteria for microscopic identification of convoluted nuclei.

**Figure 7:**
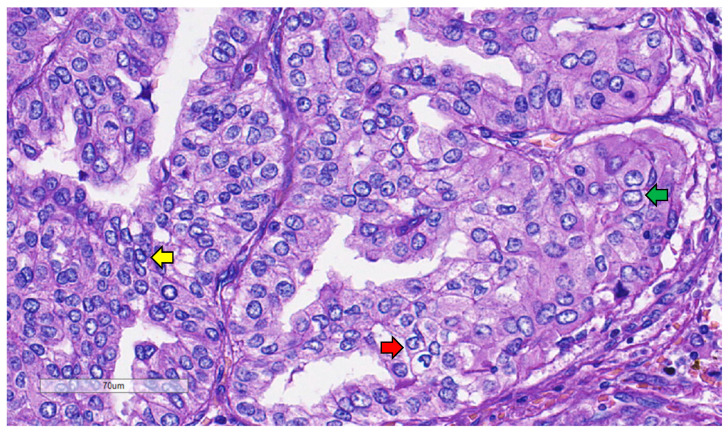
Papillary thyroid carcinoma nuclear features presented cells that overlapped (yellow arrow) with irregular nuclear membranes (red arrow) and chromatin-clearing (green arrow) (hematoxylin and eosin).

On the other hand, PTC-like nuclei are a distinct feature in diagnosing PTC. It is characterized by the presence of nuclei that appear enlarged and elongated, along with irregular nuclear contour, nuclear grooves, and chromatin clearing (Figure 7) [37]. Unlike Turin criteria, which have excluded PTC-nuclei in the diagnosis of PDTC, HHG criteria incorporate PTC-like nuclei. This discrepancy arises from the conflicting studies in which PTC-like nuclei may represent well-differentiation and good prognosis in PTC cases but not in DHGTC cases. The predominance of proposed molecular changes in DHGTC (*BRAF*V600E) might explain the preserved PTC-like nuclei in this subtype [12]. Thus, the addition of DHGTC as a new subtype of thyroid malignancies might justify this issue [1,8].

#### 3.2.5. Role of Ki-67

Immunohistochemistry (IHC) is used mainly to investigate tumors’ site of origin and possible clinical behavior. The information may help in deciding a more tailored therapeutic approach. Ki-67 is one of the commonly used IHC markers to detect cell proliferation and growth in neuroendocrine neoplasm. It originally came from the protein product of the gene *MK167* [38]. Beyond necrosis and mitosis, the role of Ki-67 has been a longstanding topic of discussion in the field of thyroid carcinoma. The combination of Ki-67, necrosis, and mitosis for predicting tumor aggressiveness warrants further elucidation [19]. In several studies, Ki-67 indices classified differentiated carcinoma into low-, moderate-, and high-risk groups, with cut-off values of <5%, 5–10%, and 10–30%, respectively. Previously, it has been an understudied marker to detect differentiation in thyroid carcinoma. As research advanced, it was proven that Ki-67 labeling indices can be used for differentiating FTC and FA, and are also significantly related to worse disease progression and prognosis in thyroid carcinoma [9,21,39,40,41]. Gnemmi et al. [9] learned the complementary prognostic role of Ki-67 cut-off of ≥4% in both PDTC cases assessed by Turin and HHG criteria [9]. However, it is still debatable whether the Ki-67 labeling index could be a tool for the diagnosis of PDTC and DHGTC, and its role in prognostic markers still yields variable results between studies [9,16].

#### 3.2.6. Cytology

Fine needle aspiration (FNA) has an important role in diagnosing thyroid neoplasms and could be considered the gold standard evaluation for initial thyroid nodules. It is a basic alternative to determine the risk of malignancy (ROM) as well as the initial management of thyroid nodules. It is crucial for preoperative evaluation to prevent over-management of the surgical approach, as it may have a long-term influence on the patient’s thyroid status, requiring continuous hormone replacement therapy. As WHO released the newest classification of thyroid neoplasms, the 2023 Bethesda System for Reporting Thyroid Cytopathology (TBSRTC) was released accordingly. It is highly specific and sensitive for screening of thyroid nodules, with a positive predictive value (PPV) of 97–99%. The PPV can be especially higher if combined with the advancement of molecular testing [42]. Following the identification of the novel entity DHGTC, experts have been diligently seeking indicators of the diagnosis in cytological specimens [43]. Previous studies by Tondi Resta et al. [21] presented the cytology report of 32 DHGTC cases, of which the majority staged Bethesda VI (14 cases). It is said to be matched with the previous surgical pathology diagnosis of mostly PTCs. The cytology findings were as follows: clusters of tumor cells with oncocytic to clear cytoplasm, pleomorphic cells, overlapping nucleus, with nuclear features such as elongation, thickened membrane, chromatin clearing, and micronucleoli [21]. Evaluating cytology specimens for increased mitotic count and necrosis is critical, especially of the karyorrhexis type, as these factors can serve as early indicators of high-grade tumors [44]. Definitive diagnosis of DHGTC in cytology specimens is challenging, as the features may also be present in rare non-invasive tumors. In addition, an infarction could also be mistaken for true tumor necrosis. However, finding these features in cytology specimens indicates a differential diagnosis of DHGTC and PDTC, and warrants further investigation [44,45].

## 4. Clinical Features

The global prevalence of PDTC and DHGTC varies, constituting less than 5% of thyroid malignancies in the United States and less than 1% in Japan [2,14,34,46]. However, these malignancies show a higher prevalence in other nations, particularly in Latin America and Europe, such as Northern Italy, where it surpasses 5% [2,14,34,46]. These differences are thought to stem from geographical and environmental influences. Higher incidences are noted in regions characterized by iodine deficiency, especially in higher altitude areas [14,34]. Additionally, there is a suggestion that these malignancies may originate de novo, possibly associated with iodine deficiency [46]. These findings imply a potential role for ethnic or dietary factors, specifically iodide intake, in the development of PDTC and DHGTC.

DHGTC and PDTC predominantly affect adults, typically in their fifth to sixth decade of life [2,14,34,46]. They present as rapidly growing masses [2]. Compared with PDTC, DHGTCs tend to be more commonly diagnosed in older patients [12,16,25]. In terms of female-to-male ratios, there are no significant differences in the diagnosis of both PDTC and DHGTC [12,14,46]. Despite this, a slight female predominance is observed in some reports of these malignancies [2,16,34].

Both DHGTC and PDTC are generally rare in children and young patients; however, descriptions of these cases, especially in teenagers, have been reported with a slight predominance of females in PDTC cases [14,46,47]. Notably, these demographic exhibits distinct clinical profiles, with cases demonstrating an aggressive disease course leading to lethal outcomes [47]. Due to the variability in diagnostic standards employed over time and the infrequency of the diagnosis in young individuals, comprehensively assessing this demographic group proves to be challenging [14,47].

An initial examination was completed using ultrasound, scintigraphy, and FDG-PET. The results show solid, heterogeneous, and hypoechoic mass with irregular and indistinct borders on ultrasound, a cold appearance in scintigraphy, and a positive result on FDG-PET [1,22]. Both PDTC and DHGTC can be found in different sites other than the thyroid glands as long as the thyroid parenchyma tissue is present, e.g., the mediastinum, thyroglossal duct cysts, and ovary. Metastasis is often found in lymphatics or hemogenic to other distant extrathyroidal sites (soft tissue, lungs, bones) [1,21]. While metastases are prevalent in both DHGTC and PDTC, occurring in approximately 20 to 50% of patients [14,46], a discrepancy in reports lies in the occurrence of distant metastases at the time of diagnosis. Initially, it was assumed that there was no discernible difference between DHGTC and PDTC in revealing distant metastases at the time of diagnosis [12,25]. However, emerging findings from more recent reports challenge this notion, favoring DHGTC in the occurrence of distant metastases at the time of diagnosis [25].

Varied reports exist regarding differences in tumor size between poorly differentiated tumors. According to Wong et al. [12], there were no notable distinctions in tumor size or the presence of distant metastases at the time of diagnosis, while other studies highlight distinct features favoring DHGTC. Specifically, DHGTC exhibited a higher frequency of characteristics, such as larger tumor size and vascular invasion [25]. Vascular invasion of more than four vessels has been linked to poor prognosis [48]. Venous obstructions are mostly associated with aggressive thyroid tumors. However, they are still considered uncommon, with only a few cases involving invasion of the neck or central veins [49]. Although assessing preoperative vein invasion is challenging, it is essential, as it plays a crucial role in determining the appropriate aggressive treatment modality to improve survival [49]. Additionally, vascular invasion has been identified as a risk factor for venous tumor thrombi in the internal jugular vein (IJV) [50,51]. Preoperative diagnosis of venous obstructions, such as neck and central vein invasion, can be accomplished using ultrasound (US) color Doppler and Doppler spectral analysis. US color Doppler with the Valsalva maneuver can help depict vascular invasion, while Doppler spectral analysis evaluates the spectral waveforms of the subclavian and internal jugular veins on both sides to determine the location, side, and severity of central vein lumen occlusion [52]. A case presented by Morvan et al. [53] described a very rare instance of a PDTC with IJV thrombus and multiple bone metastases. The patient underwent preoperative US evaluation, which showed a thyroid nodule and right IJV tumor thrombi invasion. These findings led to more aggressive tumor management. In contrast, Jafari et al. [54] presented a case report of a patient referred with a palpable neck mass one month after a total thyroidectomy, as preoperative ultrasonography failed to identify vascular involvement. This case underscores the importance of vigilant assessment with IJV ultrasound before management, as a sudden enlargement of a pre-existing nodule may indicate progression into PDTC or DHGTC.

## 5. Molecular Properties of Differentiated High-Grade Thyroid Carcinoma

The pathogenesis of thyroid carcinoma is significantly influenced by the mitogen-activated protein kinase (MAPK) and AKT (Protein Kinase B) pathways. In normal thyroid cells, the MAPK pathway plays a crucial role in regulating essential cellular functions. This pathway conveys signals from the cell surface to the nucleus by utilizing various receptor tyrosine kinases (RTKs), which are activated by extracellular ligands. Upon activation, RTKs form dimers and initiate cascades of phosphorylation events, leading to the activation of MAP kinase (MAPK) [2,55].

The activated receptor, with the involvement of adaptor proteins, triggers the activation of RAS situated on the inner face of the plasma membrane. Activated RAS recruits RAF proteins, primarily BRAF in thyroid follicular cells, to the plasma membrane. BRAF, when activated, phosphorylates, and activates the mitogen-activated protein kinase/ERK kinase (MEK), which subsequently phosphorylates and activates the extracellular-signal-regulated kinase (ERK). The activated ERK translocates to the nucleus, where it regulates the transcription of genes associated with cell differentiation, proliferation, and survival. In thyroid cancer, alterations in this pathway can occur at different levels due to point mutations or rearrangements involving the *RAS* and *BRAF* genes [2,55].

As *BRAF* and *RAS* genes play an important part in the MAPK pathway, thyroid neoplasms are molecularly categorized into two groups: *BRAF*V600E-like and *RAS*-like, or three groups: *BRAF*V600E-like, *RAS*-like, and other copy number alterations that lead to a multi-step molecular change. These primary driver mutations remain present in all thyroid carcinomas [53]. Notably, PDTCs more commonly harbor *RAS*-like driver mutations when originating from precursor follicular-patterned lesions, such as FTC or follicular variant of papillary thyroid carcinoma (fvPTC), while DHGTCs, emerging from precursor conventional PTC, frequently exhibit *BRAF*V600E-like driver mutations [13,14,15,56].

Cells harboring the *BRAF*V600E mutation experience persistent activation of the BRAF kinase, leading to continuous and autonomous stimulation of the MAPK pathway, even in the absence of extracellular ligands, contributing to the pathogenesis of thyroid carcinoma [2,55]. This abnormal activation is a crucial factor in the initiation of tumorigenesis in thyroid cells. Furthermore, beyond the early stages of tumor development, this mutation also plays a role in tumor invasion and metastasis [55].

As previously mentioned, *RAS* mutations play a significant role as a primary driver in the pathogenesis of thyroid neoplasms. The RAS protein serves as an upstream regulator of the MAPK pathway. Its distinctive capability to transition between inactive and active states, depending on ligand binding, allows it to modulate downstream signaling. In thyroid cancer with *RAS* mutations, the altered RAS protein, resulting from the point mutation, disrupts this equilibrium, remaining in an active state. This persistent activation leads to sustained stimulation of cell proliferation, impaired apoptotic processes, and the development of carcinomas [55].

**Figure 8:**
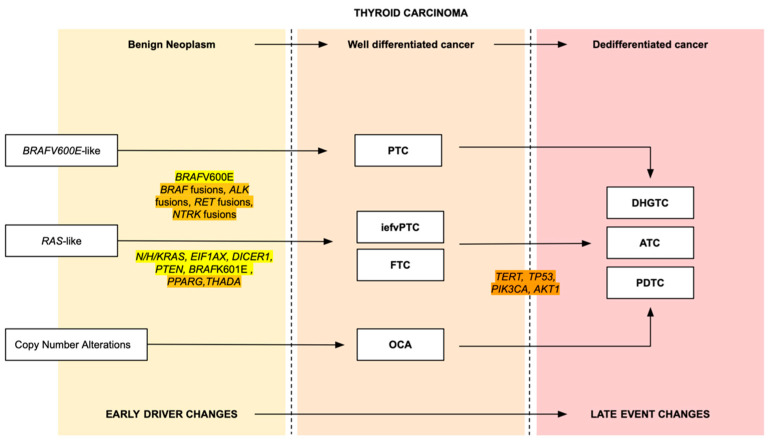
Molecular progression of thyroid carcinomas. Gene mutations are highlighted in yellow, gene fusions in light orange and late-stage molecular mutations in dark orange. Abbreviations: PTC, papillary thyroid carcinoma; iefvPTC, invasive encapsulated follicular variant papillary thyroid carcinoma; FTC, follicular thyroid carcinoma; OCA, oncocytic carcinoma DHGTC, differentiated high-grade thyroid carcinoma; ATC, anaplastic thyroid carcinoma; PDTC, poorly differentiated thyroid carcinoma.

Both *BRAF*V600E-like and *RAS*-like thyroid cancers can undergo additional genetic alterations, including gene mutations and/or gene fusions, progressing to high-grade carcinomas (Figure 8). Notably, for *BRAF*V600E-like tumor progression to DHGTC goes through the *BRAF*V600E mutation and gene fusions involving *BRAF*, *RET* (a gene that encodes RTKs), and neurotrophic receptor kinase 1 or 3 (*NTRK1/3*) [2,3,13,46]. *RAS*-like molecular profiles encompass *NRAS*, *HRAS*, *KRAS*, *EIF1AX* (eukaryotic translation initiation factor 1A, X-linked), *DICER1* (dicer ribonuclease III), phosphatase and tensin homolog (*PTEN*) mutations, *BRAF*K601E, and gene fusions involving peroxisome proliferator-activated receptor-gamma (*PPARG*) and *THADA* (thyroid adenoma associated gene) [2,3,13,46]. While the molecular characteristics of poorly differentiated carcinomas can be identified, the corresponding histological features may be absent [14].

The non-driver mutations that contribute to the late-stage thyroid tumorigenesis of PDTC and DHGTC include *TERT*, *TP53*, *PIK3CA*, and *AKT1* [2,15,56]. Specifically, *TP53* and *TERT* promoter mutations are the two main genetic alterations in PDTC and DHGTC [2,3,12,13,46,57]. These late-stage mutations, especially TERT mutations, indicate aggressiveness and are recognized as significant independent prognostic factors [25]. These mutations are rarely observed in well-differentiated thyroid carcinomas, with frequencies increasing incrementally from well-differentiated thyroid carcinomas to ATC [58]. The rate of *TERT* promoter mutations is comparable between PDTC (44–59%) and DHGTC (39–52%), whereas *TP53* mutations are more frequent in PDTC (16%) compared to DHGTC (4%) [15]. Six percent of PDTCs also harbor *PIK3CA* mutations [15].

The persistence of driver mutations like *BRAF* and *RAS*, alongside the increasing occurrence of additional alterations such as *TERT* promoter mutations, *TP53* mutations, and changes in the PIK3CA-AKT-mTOR pathway, highlights a stepwise molecular progression from well-differentiated to ATC (Figure 8) [2,15,56]. These findings underscore the complex molecular landscape and progression in thyroid cancer, providing valuable insights for understanding its pathogenesis and informing clinical management.

## 6. Treatment and Management

Since PDTC and DHGTC were classified into a category mirroring intermediate disease progression and prognosis, they require more aggressive treatment and follow-up. Several differences regarding molecular alterations between PDTC and DHGTC marked not only the distinct pathological features but also the response to treatment. Recent studies have showed about 50% of PDTCs possess mutations in pathways targeted by drugs [14,46]. This nuanced understanding of tumor characteristics and progression underscores the complexity of managing PDTC and DHGTC, emphasizing the importance of tailored management strategies.

At early stages, complete resection can be effective and show a good prognosis. However, adjuvant therapies might be needed in more advanced stages with/without distant metastases [12,13,16,21]. Considering the new subtype has just been introduced in the practice of TC diagnosis, there is still limited data regarding treatment recommendation and follow-up.

The mainstay of treatment for PDTC is total thyroidectomy with lymph node dissection. Initial surgical staging is essential for disease management and prognosis. Other procedures like esophageal submucosal resection, unilateral recurrent nerve resection, or palliative surgery could be completed in terms of tumor invasion to the surrounding structures (esophagus, trachea, larynx, recurrent nerve) to lower the risk of the compartment in the neck, to prevent a locoregional recurrence, and other life-threatening conditions such as airway obstruction and hemorrhage [59,60,61].

Adjuvant therapies might be needed in cases such as metastases and unresectable tumors. Some retrospective cohort studies showed a variety of modalities used for the treatment in both PDTC and DHGTC. This includes radioactive iodine (RAI), external beam radiation therapy (XRT), chemotherapy, and a combination of RAI-XRT, or chemo-XRT. The standardized therapeutic approach for PDTC and DHGTC has not been established and remains understudied [16,21,59,60,61].

Generally, post-operative thyroid cancers are monitored using radioactive iodine or serum thyroglobulin. However, in the case of PDTC and DHGTC, this approach is often ineffective. Due to their lower avidity for radioactive iodine, both PDTC and DHGTC can develop as radioiodine-refractory thyroid carcinoma [2,14,46,62,63]. Another method of monitoring recurrence is through thyroglobulin; however, in accordance with their histologic and immunophenotypic profile, PDTCs may not secrete appreciable quantities of thyroglobulin [14,46,62]. These challenges emphasize the need for different surveillance strategies. Currently, it is recommended to use Fluorodeoxyglucose-Positron Emission Tomography (FDG-PET) scans for monitoring, both in the initial staging processes and follow-up after treatments [14,46,62].

## 7. Prognosis

Despite their rarity, both DHGTC and PDTC stand as intermediate prognostic forms within the spectrum of thyroid cancer. They demand precise diagnosis, effective treatment, and vigilant follow-up, considering that 44% of patients die of the disease. The overall prognosis of DHGTC aligns closely with that of PDTC. Reported five- and ten-year OS rates for PDTC range from 62% to 85%, with corresponding disease-specific survival (DSS) rates of 66% and 50%, respectively [2,46,62]. In cases meeting PDTC or HGDTC criteria, the long-term survival was discouraging, with 3-year, 5-year, 10-year, and 20-year OS rates of 88%, 75%, 54%, and 28%, respectively, along with DSS rates of 89%, 76%, 60%, and 35% [46,63]. When looking at the diagnosis of HGDTCs alone, they exhibit a comparable disease-specific survival of 56% at ten years [2,46].

Several factors have been identified to affect patient prognosis. We can divide these factors into clinical factors, morphologic/histologic factors, and molecular factors. Patients typically present with a clinical diagnosis at an older age (greater than 45 years), often accompanied by advanced local-regional disease and metastasis [14,46,57]. Common sites of metastasis were the lungs, bone, and brain [63]. Macroscopically, a sizable tumor size of ≥5 cm [57], noticeable extrathyroidal extension, and the presence of distant metastases are observed [14,46,56,57]. Extrathyroidal extension and distant metastasis may act as independent risk factors contributing to a poorer prognosis [46]. Positive surgical margins and nodal metastasis during primary resection are also recognized as independent pathologic prognostic factors [56].

Minor histologic components arising from either PDTC or DHGTC within the context of a differentiated thyroid carcinoma can influence the prognosis [14]. When the poorly differentiated tumor volume comprises around 10% of the total, the overall prognostic implications resemble those of predominantly poorly differentiated lesions and are associated with a poor prognosis [14,57]. This finding is similar to tumors with over 50% PDTC [15]. We know that both PDTC and DHGTC diagnoses include the findings of tumor necrosis. The extent of tumor necrosis significantly affects DSS, distant metastasis-free survival, and locoregional recurrence-free survival [63].

Certain microscopic characteristics beyond the diagnosis of PDTC and DHGTC hold clinical significance. Evidence suggests that not only the presence of capsular/vascular invasion but also the extent of such invasion carry prognostic relevance for PDTC [14,15,46]. The 5-year disease-free survival varied across different scenarios: 83% in patients with tumors exhibiting focal capsular invasion, 100% in tumors with focal vascular invasion, 57% in tumors with extensive vascular invasion (4 or more foci of vascular invasion), and 10% in widely invasive tumors [64]. If we stratified the patients who were M0 at diagnosis, the outcomes showed slight improvement with a 5-year DFS of 100% in tumors with focal invasion, 73% in tumors with extensive vascular invasion, and 17% in widely invasive disease [64]. Additionally, studies indicate that encapsulation is linked to enhanced survival and reduced risk of recurrence in patients with DHGTC as well [56]. Therefore, pathologists need to meticulously document the extent of PDTC or DHGTC characteristics, tumor necrosis, encapsulation, extent of vascular invasion, and the presence of extrathyroidal extension, in addition to pathologic TNM staging. This comprehensive documentation of findings is essential to assist clinicians in evaluating prognosis and guiding appropriate treatment strategies.

Cases of PDTC in children constitute a clinicopathological and molecular entity that can exhibit aggressive behavior. This is contrary to expectations based on their mutational profile. Unlike those observed in older adults, *DICER1* mutation found in pediatric PDTC is often associated with aggressive disease behavior [14,46,47]. These genetic variations influence the clinical course, with only some cases demonstrating low-risk behavior [46]. Moreover, these tumors often contain a component of well-differentiated PTC, with some instances displaying encapsulation, a feature linked to a more favorable prognosis in adults [47]. Generally, pediatric DHGTC tends to have a less aggressive course compared to its adult counterpart [56]. Nonetheless, distant metastasis and, exceptionally, disease-related mortality may also occur in this age group [56].

## 8. Conclusions

In conclusion, DHGTC has been recently recognized as a distinct entity within the spectrum of thyroid carcinomas. This tumor is defined as an invasive tumor of PTCs/FTCs/OCAs with ≥5 mitoses per 2 mm^2^ and/or tumor necrosis with no anaplastic features. It occupies an intermediate prognostic position, resembling that of PDTC, and is stratified between well-differentiated and undifferentiated thyroid carcinomas. A thorough pathology examination is imperative for accurate diagnosis. Moreover, discrepancies in mitotic thresholds between DHGTC and PDTC warrant further clarification. Accounting for its aggressive behavior, accurate diagnosis and recognition by pathologists are imperative. These recent updates have given a promising environment to elaborate the conventional way of diagnosis with meticulous morphological features, together with clinical and molecular points of view.

## Figures and Tables

**Table 1:** World Health Organization Diagnostic Criteria of Poorly Differentiated Thyroid Carcinoma and Differentiated High-Grade Thyroid Carcinoma [1].

Criteria	PDTC	DHGTC
Growth Pattern	Solid/insular/trabecular	Papillary/follicular/solid
Invasion	Yes	Yes
Papillary nuclear features	No	Yes/no
High-grade features	Mitotic count ≥ 3/2 mm^2^ or tumor necrosis or convoluted nuclei	Mitotic count ≥ 5/2 mm^2^ or tumor necrosis
Well-differentiated carcinoma component	No or retention of some	Yes (PTC/FTC/OCA)
Anaplastic features	No	No

Abbreviations: PDTC, poorly differentiated thyroid carcinoma; DHGTC, differentiated high-grade thyroid carcinoma; PTC, papillary thyroid carcinoma; FTC, follicular thyroid carcinoma; OCA, oncocytic carcinoma of the thyroid.

**Table 2:** Previous Studies in The Diagnosis of Differentiated High-grade Thyroid Carcinoma.

First Author, Year	Jeong S.I. et al., 2023 [25]	Tondi Resta I. et al., 2024 [21]	Thompson et al., 2023 [16]
Study type	Cohort	Cohort	Cohort
Country	Republic of Korea	USA	USA
Collection time	May 2019–December 2021	2012–2022	January 2010–December 2021
Number of cases	14	32	17
Cytology (TBSRTC)	N/A	Yes	N/A
I		1	
II		0	
III		5	
IV		10	
V		2	
VI		14	
Necrosis			
Present	11	21	17
Absent	3	11	0
Mitosis (mean/2 mm^2^)	3.14	3.2	6.1
Invasion			
Lymphatic	12	21	8
Vascular	3	6	14
PNI		1	
None		8	
ETE			
Yes	8	17	4
No	6	15	13
Ki 67 labeling (median)	N/A	3.50%	8.30%
Metastases			
Regional		4	
Nodes	12	10	2
Organ/distant	2	5	4
Last follow-up status	No evidence of disease (12) Alive with disease (2)	Alive without dx (24)	No evidence of disease (11)
		Alive with dx (1)	Alive, with metastatic disease (2)
		Died from dx (1)	Dead, with no evidence of disease (1)
		Died from other causes (1)	Dead, with metastatic disease (3)
		Lost to follow-up (5)	
Number of deaths	0	1	4
Treatment	N/A	RAI (18)	Surgery only (4)
		RAI + other chemotherapy (2)	RAI (11)
		RAI + XRT (1)	External beam radiation (3)
		Chemotherapy (1)	Chemotherapy (4)
		Chemotherapy + XRT (1)	
		No additional therapy (4)	

Abbreviations: TBSRTC, the Bethesda system for reporting thyroid cytopathology; PNI, perineural invasion; ETE, extrathyroidal extension; RAI, radioactive iodine; XRT, external beam radiotherapy.
